# Happy Hypoxia With SARS-CoV-2 Pneumonitis: A Life-Threatening Case of a Medical Doctor

**DOI:** 10.7759/cureus.80181

**Published:** 2025-03-06

**Authors:** Jeevan K Sharma, Padmini Yadav, Rahim R Sewani, Jyoti Sharma, Prashant Tripathi

**Affiliations:** 1 Spine Surgery, Indian Spinal Injuries Center, New Delhi, IND; 2 Surgery, All India Institute of Medical Sciences, New Delhi, New Delhi, IND; 3 Family Medicine, Evershine Med Bedford Family Medicine, Euless, USA; 4 Pediatrics, Kanti Children's Hospital, Kathmandu, NPL; 5 Internal Medicine, Grande International Hospital, Kathmandu, NPL

**Keywords:** case report, happy hypoxia, medical doctor, pneumonitis, sars co v2

## Abstract

The second wave of severe acute respiratory syndrome coronavirus-2 (SARS-CoV-2) had devastating consequences in developing countries like Nepal and India. SARS-CoV-2 affected everyone, encompassing all professions, age brackets, and socioeconomic levels. Symptoms, along with the SARS-CoV-2 reverse transcription-polymerase chain reaction (RT-PCR) results, were the primary factors used to determine whether patients should be instructed to quarantine at home or be admitted to hospitals immediately. Happy hypoxia refers to a situation where a patient shows no respiratory symptoms yet experiences a decline in oxygen saturation levels. This drop can go unnoticed, potentially resulting in a sudden deterioration of the patient’s condition, with reports of fatalities occurring. We present a case involving a physician who experienced a sore throat and fever as symptoms but had no respiratory distress, along with a decrease in oxygen saturation to 87% at home and 82% at the hospital, resulting in a near-fatal situation.

## Introduction

The second wave of severe acute respiratory syndrome coronavirus-2 (SARS-CoV-2) in India and Nepal from March to June 2021 took a great toll on the livelihood of people. It led to devastating consequences like overburdening of the health system, roadways being blocked, and scarcity of oxygen supplies [1]. SARS-CoV-2 pneumonitis affects the lungs, leading to fluid collection and alveolar destruction, finally leading to a devastating complication known as acute respiratory distress syndrome (ARDS) with a high fatality rate. ARDS is an inflammatory process where there is endothelial damage in lung tissues leading to fluid accumulation warranting immediate attention for intensive care of the patient for its recovery [2]. Silent hypoxemia is another term for happy hypoxia. It is a phenomenon in which a patient has minimal respiratory symptoms, but oxygen saturation is decreased [3,4]. Poor outcomes have been reported in patients with happy hypoxia. Though patients can worsen with happy hypoxia, recovery is also quick if they are identified early and managed accordingly [5]. We report a case of a medical doctor who was himself baffled with happy hypoxia and presented with decreased oxygen saturation (87% at home and 82% at the hospital) but no respiratory symptoms. 

## Case presentation

A 33-year-old physician, diagnosed with reverse transcription-polymerase chain reaction (RT-PCR)-positive SARS-CoV-2 on April 24, 2021, reported mild fever and a sore throat for three days. RT-PCR is a process of detecting the virus in the laboratory from a nasal swab, and if it comes back positive, the patient is deemed infected with the virus. He was not having many symptoms other than a high fever, but his home pulse oximetry reading showed 87%, for which he rushed to the hospital. Upon examination, his pulse was recorded at 114 beats per minute, blood pressure measured 110/70 mm Hg, respiratory rate was 20 breaths per minute, oxygen saturation was 82% in ambient air and improved to 97% when on oxygen, and his temperature was 98 degrees Fahrenheit. Auscultation of the chest revealed bilateral crackles. The examination of other systems showed no remarkable findings, and there were no indications of respiratory distress.

The patient was admitted to the Intensive Care Unit (ICU) on April 27, 2021, with the aforementioned symptoms. The patient underwent evaluation and investigations. Initial blood tests indicated an elevated neutrophil count and a reduced lymphocyte count. Additionally, there were increased levels of C-reactive protein (CRP), lactate dehydrogenase (LDH), ferritin, D-dimer, interleukin-6 (IL-6), procalcitonin, and N-terminal pro-B-type natriuretic peptide (NT-pro-BNP) (Table 1).

**Table 1:** Initial blood reports of the patient at the time of admission ESR: erythrocyte sedimentation rate; CRP: C-reactive protein; LDH: lactate dehydrogenase; PT: prothrombin time; INR: international normalized ratio; NT-Pro BNP: N-terminal pro-B-type natriuretic peptide

Test	Patients value	Reference value
Total leucocyte count (n/cu.mm)	6,580	4,000-11,000
Neutrophils (%)	91.2	40-60
Lymphocytes (%)	7.4	20-40
Monocyte (%)	0.9	2-8
Eosinophil (%)	0.2	<5
Basophil (%)	0.3	0.5-1
Platelet count (lakh/cu.mm)	1.69	1.5-4.5
ESR (mm/1^st^ hour)	13	0-15
CRP (mg/dL)	189.62	<1
LDH (U/L)	467.0	135-225
Ferritin (ng/ml)	930.80	30-400
D-dimer (ng/ml)	2708.7	<500
PT (sec)/INR	12.7/0.94	11-13.5/0.8-1.1
IL-6 (pg/mL)	47.70	<4.4
Procalcitonin (ng/ml)	4.55	<0.5
NT- Pro BNP (pg/ml)	311.2	<300

**Figure 1:**
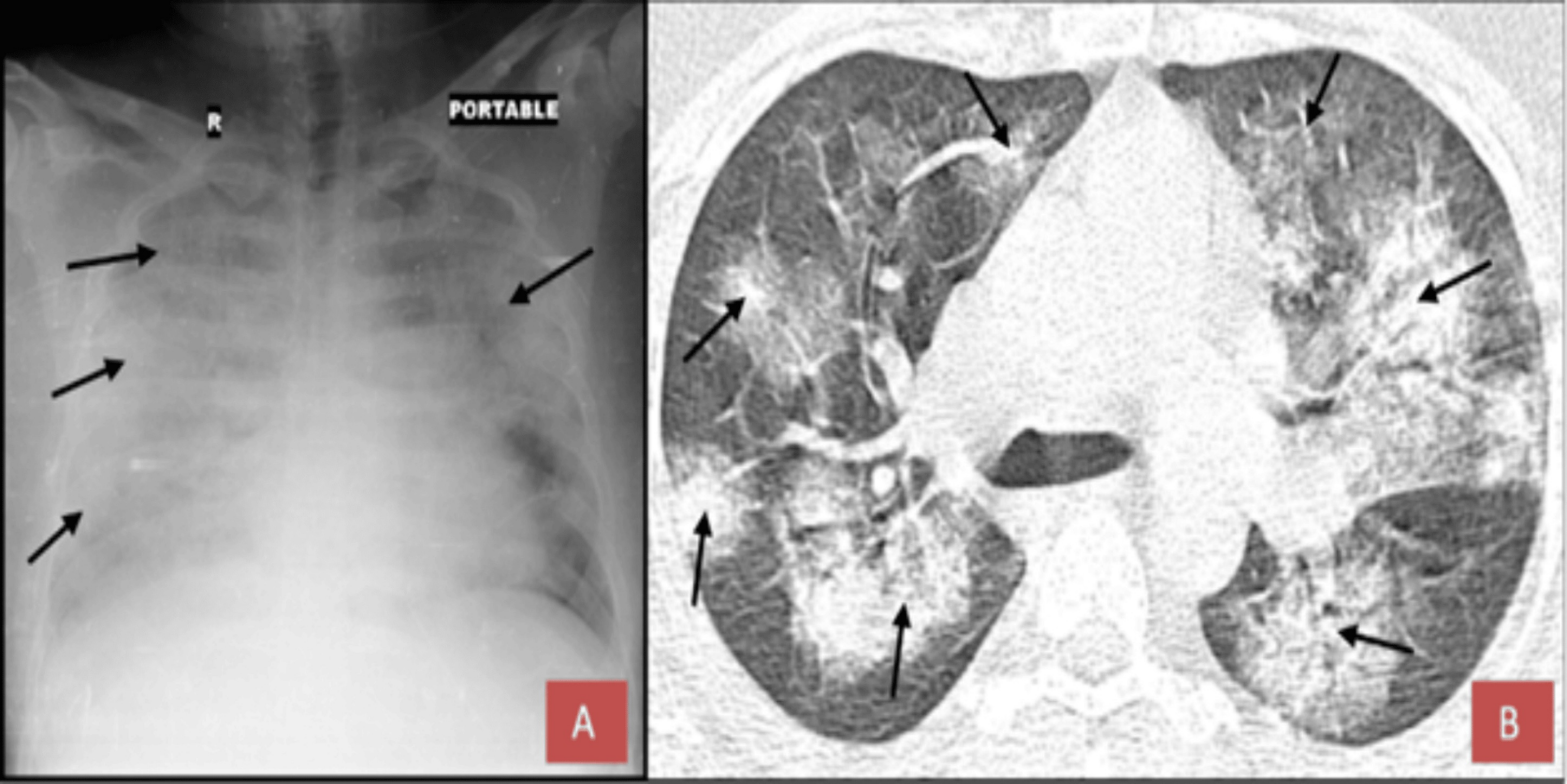
Findings X-ray (A) and CT scan (B) were performed, revealing a CT severity score of 23/25 upon admission. Arrows indicate regions of pneumonitis accompanied by pulmonary edema, leading to a complete whiteout of the lung.

The chest X-ray and high-resolution computed tomography (HRCT) of the chest revealed ground-glass opacities with a computed tomography (CT) severity score of 23 out of 25 (Figure 1).

**Figure 2:**
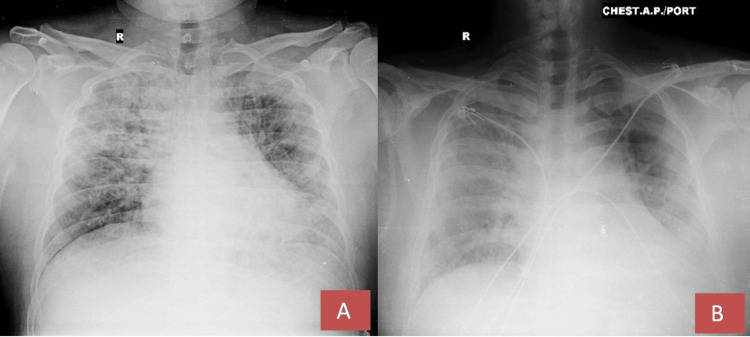
Chest X-ray findings A portable chest X-ray taken during the first week (A) and the second week (B) indicates a reduction in the radiological signs of pulmonary edema compared to the X-ray taken at the time of presentation.

Other differential diagnoses like pneumonia and pulmonary embolism were ruled out from clinico-radiological and laboratory findings. The patient received regular monitoring and was treated with non-invasive ventilation (NIV) using bilevel positive airway pressure (BiPAP) while on high-flow oxygen of 14 liters per minute, with a fraction of inspired oxygen (FiO₂) of 100% for 48 hours. Afterward, a single dose of injection (inj.) tocilizumab at 80 mg/kg (milligrams per kilogram) was administered, along with two units of plasma therapy and a loading dose of inj. remdesivir was infused intravenously at 200 mg over a period of 30-120 minutes, followed by a maintenance dose of 100 mg intravenously each day before noon for a total of five days. Inj. methylprednisolone at 2 mg/kg was delivered in two divided doses. The patient was also given enoxaparin at a dosage of 6000 international units (IU) every 12 hours. Daily morning assessments included arterial blood gas (ABG) analysis and chest X-rays to monitor the patient's condition. Additional supportive care comprised metered dose inhaler (MDI) budesonide, levosalbutamol, oral zinc, and vitamin C tablets, along with chest physiotherapy. After two days, the FiO₂ was gradually reduced to 40%, and oxygen was provided at 10 L/min (liters/minute), achieving an oxygen saturation of 96% during the first week. The patient demonstrated clinical and radiological improvement thereafter, with an increase in oxygen saturation as the FiO₂ and oxygen flow rates were decreased, ultimately reaching an oxygen saturation of 94% on room air by the tenth day of hospitalization (Figure 2).

The RT-PCR test for SARS-CoV-2 returned negative on May 10, 2021. Following this, the patient was transferred from the ICU to the inpatient ward and was discharged after a total of 16 days in the hospital (12 days spent in the ICU and four days in the ward). Blood tests conducted during the first to fourth weeks indicated a reduction in total leucocyte count (TLC) and a decrease in neutrophil count alongside an increase in lymphocyte levels. There was a steady decline in acute phase reactants, demonstrated by lower levels of ferritin, IL-6, ESR, and CRP. By the fourth week, an adequate level of SARS-CoV-2 IgG antibody titer was observed (Table 2).

**Table 2:** Laboratory results for the patient's blood at the conclusion of both the first and fourth week of treatment ESR: erythrocyte sedimentation rate; CRP: C-reactive protein

Test	Patients report at 1^st^ week	Patients report at 4^th^ week	Reference value
Total leucocyte count (n/cu.mm)	12,960	6,380	4,000-11,000
Neutrophils (%)	88.5	67	40-60
Lymphocytes (%)	6.9	21.5	20-40
Monocyte (%)	4.4	8.5	2-8
Eosinophil (%)	0.0	0.3	<5
Basophil (%)	0.2	0	0.5-1
Platelet count (lakh/cu.mm)	2.73	2.13	1.5-4.5
ESR (mm/1^st^ hour)	08	07	0-15
CRP (mg/dL)	38.44	4.23	<1
Ferritin (ng/ml)	1149	383.2	30-400
D-dimer (ng/ml)	2716.3	360	<500
IL-6 (pg/mL)	<2.7	-	<4.4
SARS-COV-2 IgG antibody titer (AU/ml)	-	105	>15

**Figure 3:**
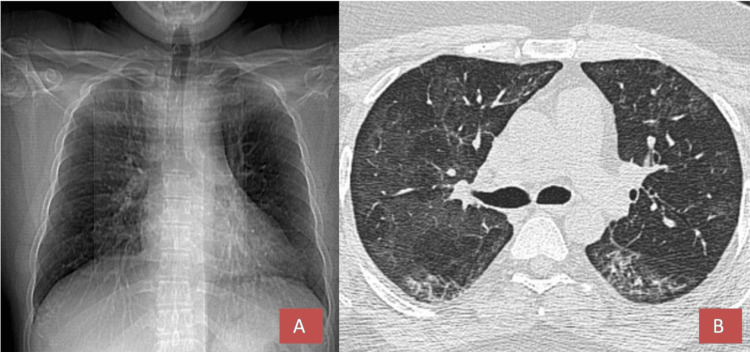
High-resolution computed tomography findings The HRCT scout image of the chest (A) in axial view (B) at the four-week follow-up indicates a reduction in pneumonitis and pulmonary edema.

Upon discharge, the patient was on a tapering regimen of tab methylprednisolone, reducing from 24 mg to 4 mg every four days, as well as taking tab apixaban 2.5 mg twice daily and tab perfenidone 200 mg every eight hours, along with supportive treatments of vitamin C and zinc. The six-minute walk test indicated a pre- and post-test oxygen saturation of 96%, with no notable symptoms or decrease in saturation, alongside an improvement in the CT scan (Figure 3).

**Figure 4:**
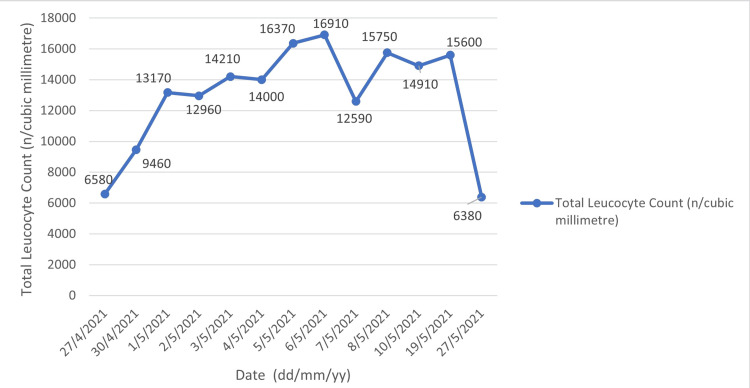
Trend of total leucocyte count for the patient from the time of admission to one month of follow-up

**Figure 5:**
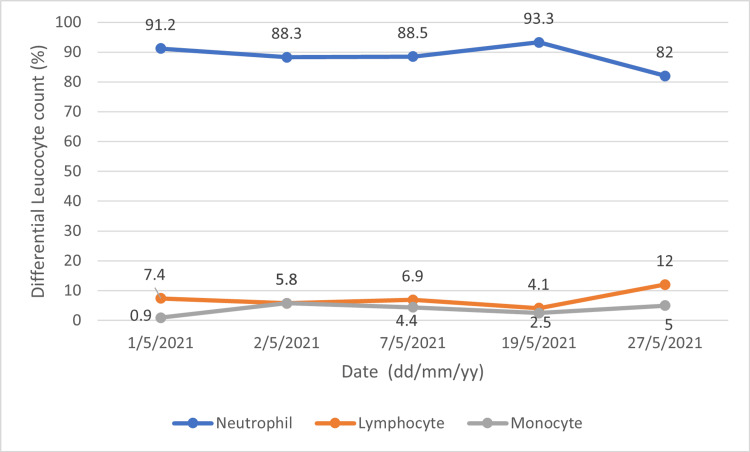
Trend of differential leucocyte count for the patient from the time of admission until one month of follow-up

The trend of total leucocyte count and differential leucocyte count (DLC) over the course of one month is illustrated below (Figures 4, 5).

## Discussion

Happy hypoxia/hypoxemia illustrates a range of SARS-CoV-2 pneumonitis where the patient experiences few signs and symptoms of respiratory distress despite having reduced oxygen saturation or partial pressure of oxygen. It has been observed that patients experiencing happy hypoxia tend to have a worse prognosis if early detection and management of the issues are not addressed properly [6].

Numerous pathophysiological mechanisms and hypotheses have been suggested to explain the phenomenon of happy hypoxia in SARS-CoV-2 pneumonitis. Dhont et al. identified several potential contributors to hypoxemia in patients who do not exhibit significant respiratory distress, including intrapulmonary shunting, impaired regulation of lung perfusion from hypoxic pulmonary artery vasoconstriction, microthrombi in the bloodstream, and diminished diffusion capacity in the lungs [7]. While there has been criticism regarding the role of microthrombi in causing hypoxemia, the use of enoxaparin has become a standard treatment for patients with elevated D-dimer levels, particularly those admitted to the ICU [8]. Duarte et al. proposed that autonomic interoception could be a factor leading to happy hypoxia; however, cadaver studies indicated minimal involvement of the vagus or glossopharyngeal nerves [9]. Swenson et al. have discussed the potential causes of happy hypoxia to include vasoplegia, as well as abnormal sensing of oxygen levels both peripherally and centrally due to the direct effects of the virus [10]. UR et al. suggested a neural hypothesis, proposing that the virus could travel to the nucleus tractus solitarius via the vagus and glossopharyngeal nerves, resulting in various symptoms, with happy hypoxia being one of them [11]. According to Wenzhong et al., in instances of happy hypoxia, there is a compromise in hemoglobin, and the immune cells are affected by the production of reactive oxygen species [12].

The laboratory assessment of our patient aligns with the initial worsening characterized by elevated TLC, reduced lymphocyte levels, and increased LDH, CRP, IL-6, procalcitonin, ferritin, and D-dimer. Mardani et al. identified elevated LDH, CRP, alanine aminotransferase (ALT), and neutrophils as indicators of SARS-CoV-2 infection in their research [13]. In a systematic review of 30 articles from PubMed, Kermali et al. concluded that low lymphocyte and platelet counts are indicative of a severe presentation of SARS-CoV-2 infection [14]. Terpos et al. have similarly noted that decreased lymphocyte levels, along with elevated LDH, CRP, IL-6, procalcitonin, ferritin, and D-dimer, frequently occur in patients with SARS-CoV-2 infection [15]. In the patient described above, there was also an increase in lymphocyte count and a decrease in neutrophil count, TLC, IL-6, ferritin, CRP, and D-dimer, coinciding with the patient’s recovery.

Tocilizumab, despite being in the early stages of randomized controlled trials to demonstrate its effectiveness against severe SARS-CoV-2 infection, has displayed lower mortality rates and quicker recovery in a few other studies. Acting as an IL-6 receptor antagonist, tocilizumab has a reasonable mechanism of action by mitigating the effects of the IL-6 surge in the body, which underpins its application in SARS-CoV-2 treatment [16]. Our patient also exhibited significant improvement, transitioning from BiPAP to a high-flow mask within two days. There was a rapid decline in IL-6 level in one week. It might be due to tocilizumab being an IL-blocker in addition to the decrease in the inflammatory processes leading to a decrease in acute phase reactants and chemical mediators in a course of treatment.

## Conclusions

In summary, happy hypoxia can be misleading not only for the general public but also for healthcare professionals when assessing a patient. Timely identification, proactive management, and immediate treatment with the current standard therapies, such as tocilizumab, remdesivir, and plasma therapy, alongside diligent monitoring and maintaining adequate oxygen levels, can be critical for survival. Additionally, it is essential to document the support required by the patient, particularly when dealing with severe SARS-CoV-2 cases. The contributions of physicians, nurses, caregivers, physiotherapists, psychologists, nutritionists, family members, and social support, as well as fostering self-belief to enhance morale, are undeniably important.
